# The Classification of Hysteria and Related Disorders: Historical and Phenomenological Considerations

**DOI:** 10.3390/bs5040496

**Published:** 2015-11-06

**Authors:** Carol S. North

**Affiliations:** 1 Department of Psychiatry, The University of Texas Southwestern Medical Center, 6363 Forest Park Road, Dallas, Texas, TX 75390, USA; E-Mail: carol.north@utsouthwestern.edu; Tel.: +1-214-648-5375; Fax: +1-214-648-5376; 2 The Altshuler Center for Education & Research, Metrocare Services, 1380 River Bend Drive, Dallas, TX 75247, USA; Tel.: 1-214-743-1200

**Keywords:** dissociation, conversion, somatization, borderline personality disorder, hysteria, diagnostic classification, Briquet’s syndrome, nosology, diagnostic comorbidity, mental disorders

## Abstract

This article examines the history of the conceptualization of dissociative, conversion, and somatoform syndromes in relation to one another, chronicles efforts to classify these and other phenomenologically-related psychopathology in the American diagnostic system for mental disorders, and traces the subsequent divergence in opinions of dissenting sectors on classification of these disorders. This article then considers the extensive phenomenological overlap across these disorders in empirical research, and from this foundation presents a new model for the conceptualization of these disorders. The classification of disorders formerly known as hysteria and phenomenologically-related syndromes has long been contentious and unsettled. Examination of the long history of the conceptual difficulties, which remain inherent in existing classification schemes for these disorders, can help to address the continuing controversy. This review clarifies the need for a major conceptual revision of the current classification of these disorders. A new phenomenologically-based classification scheme for these disorders is proposed that is more compatible with the agnostic and atheoretical approach to diagnosis of mental disorders used by the current classification system.

## 1. Introduction

The relationships among dissociative, somatoform, and conversion disorders have long been uncertain and uneasy in the history of efforts to classify and understand them. The current classification of these disorders has evolved over centuries from common historical roots in a syndrome previously known as hysteria that has been interlinked in some periods with spiritual maladies. Uncertainties surrounding the origins and classification of these disorders have generated recurrent, often heated controversies among clinicians and academicians in different eras. The controversies blaze on today. This article will summarize the history of the conceptualization of these phenomena in relation to one another, chronicle efforts to classify these and other phenomenologically-related psychopathology in the American diagnostic system for mental disorders, and trace the subsequent divergence in opinions of dissenting sectors on classification of these disorders. Finally, this article will consider the extensive phenomenological overlap of all these disorders in empirical research, and from this foundation will present a new model for the conceptualization of these disorders.

## 2. A Long History of Dissociation, Conversion, and Hysteria

Dissociative phenomena have been a recognized part of human history for a very long time. Written records from ancient Egypt described cases of spirit possession, which in retrospect have been interpreted as dissociative phenomena [1]. Evidence of dissociation was also recorded in Christian scripture. Biblical passages in Mark 5:1–20 [2] describe a man possessed with unclean spirits who lived in a cemetery, injured himself with stones, and broke all chains used to restrain him. When Jesus asked his name, the man said, “My name is Legion, for we are many.” Jesus transferred the unclean spirits into a herd of swine that ran off a cliff and drowned in the sea. This story has been interpreted as representing a case of dissociative identity disorder, successfully cured with exorcism [3].

Through the end of the eighteenth century, spirit possession remained a dominant explanation for experiences of altered states of identity, and cases emerged in which the person’s self had been taken over or “possessed” by demons or evil spirits. Accounts of spirit possession in this period included descriptions of dramatic hysterical and hypochondriacal presentations and convulsive fits [4]. The practice of exorcism of demons and evil spirits came to dominate as the preferred treatment for such problems around this time. Consistent with these trends, occult fads such as table-tipping, spirit-rapping, divining, spirit séances, and use of Ouiji boards to communicate with the dead began in the United States in the nineteenth century and increased in popularity that spread to other parts of the world.

In 1646, a woman tavern owner was reported to have alternate personalities, representing history’s first clearly described case of this syndrome. This case was diagnosed by a German-Swiss alchemist/astrologer known as Paracelsus, whose birth name was Philippus Aureolus Theophrastus Bombastus von Hohenheim. Though Paracelsus claimed to be a physician, his doctoral degree in medicine could never be located [5,6]. Additional case reports of alternate personalities cropped up over the next two centuries, many of which had prominent hysterical features with dramatic physical and neurological symptoms. Increasingly bizarre cases of alternate personalities and flamboyant hysterical presentations captured the medical profession’s attention, diverting interest from the previously popular concepts of spirit possession.

Approaches to treatment of altered states of experience of self began to evolve as physicians came to consider these cases to be medically rather than spiritually based [7]. Paracelsus advanced an elaborate theory of magnetism in the human body and its role in medical illness [8]. About a century after Paracelsus, Franz Anton Mesmer incorporated this theory into a new approach to treat medical disease, based on the notion that tidal influences of the planets exert a universal magnetic force on humans, which he called “animal magnetism.” His patients were predominantly women, many of whom presented with prominent hysterical features. Mesmer’s magnetism treatment was applied with a magnet (which he realized was not the therapeutic element) in conjunction with other therapeutic techniques including mental imaging, hand gesturing, and touch methods. His methods induced states of anesthesia, paralysis, and hysterical convulsions in his patients. Mesmer was apparently quite a showman in demonstrations of these dramatic states and their cures in his patients. His critics were convinced that influences of suggestion and social contagion played a central role in the emergence of the hysterical presentations that emerged, and many considered him a charlatan [8].

Mesmer’s practice of magnetism, later termed mesmerism, eventually evolved into the modern practice of hypnosis [5]. Hypnotism, like magnetism and mesmerism, was also extensively applied in treating hysterical syndromes. The use of these methods was sometimes observed to result in the emergence of separate personalities within individuals [9]. Fascination with magnetism is thought to have further contributed to the growing popularity of occult practices and charlatanism in the nineteenth century [10].

The French psychiatrist Pierre Janet is credited with having first coined the term dissociation, borrowing the concept from an earlier conceptualization of hysterical seizures by Moreau de Tours [11]. A student of Charcot, Janet developed his theory of dissociation based on his work with patients with hysteria [11]. Janet considered dissociation to represent abnormal splitting of mental processes resulting in compartmentalization of the personality into segments inaccessible to one another [12,13,14,15]. Around the turn of the twentieth century, a surge of interest in dissociative syndromes coincided with Janet’s work, with the eruption of a small epidemic of multiple personality disorder cases that in turn elicited robust responses of professional criticism and skepticism [14]. The fascination with these cases was short-lived. In the first half of the twentieth century, attention to dissociative disorders dwindled to the point of near extinction of the syndrome. Dissociative syndromes were conceptually subsumed into hysteria, the forerunner of the modern diagnosis of somatization disorder [14].

The history of dissociation has evolved in the long shadow of the history of the much older concept of hysteria. Written records from ancient Egypt dating back at least 4000 years described a syndrome known as hysteria, which was characterized as manifestation of multiple physical and behavioral dysfunctions [16,17]. Apparently hysterical and dissociative syndromes had much in common: in the Middle Ages, syndromes considered to be hysterical in previous eras came to be conceived as products of witchcraft, demon possession, and sorcery that also had historical associations with dissociative phenomena [17,18]. The term hysteria, derived from the Greek word hystera (signifying the uterus), dates back to at least the time of Hippocrates, when it was thought that the uterus became physically displaced from its normal position in the pelvis, wandering throughout the body to create symptoms in the various places that it inhabited [19,20]. In 1697, the English physician Thomas Sydenham conceived of hysteria as an emotional condition rather than as a physical disorder, moving the source of the disorder from the uterus to the central nervous system [5,18]. Sydenham referred to hysteria as “Proteus,” acknowledging this disorder’s proclivity to simulate almost any disease [19,21,22].

In the nineteenth century, the French physician Paul Briquet used a syndromic approach defining hysteria operationally as a chronic disorder characterized by the presentation of many medically unexplained symptoms in the body’s multiple organ systems, similar to Syndenham’s earlier conceptualization of the disorder [21,23,24]. Briquet conducted a detailed systematic clinical investigation of 430 cases he evaluated over a ten-year period at the Hôpital de la Charité in Paris [23]. His 1859 article was hailed as a landmark study that “probably has no equal, either before or since it was published” [21] (p. 1401). The symptoms presented by his patients included physical complaints about bodily functions, neurological symptoms such as amnesia, paralysis, anesthesia, pain, spasms, and convulsive fits [21].

Later in the late nineteenth century, the French neurologist Jean-Martin Charcot studied young poverty-stricken rural women at a large asylum, La Salpêtrière Hospital [25,26]. His patients exhibited dramatic physical symptoms such as hysterical vomiting and neurological symptoms including anesthesia, paralysis, deafness, bizarre movements, and epileptic-like seizures [25,27]. Charcot used hypnosis with his patients. He concluded that only hysterics could be hypnotized [19]. Charcot noted striking similarities in symptom presentations among cases of hysteria and demon possession, especially the occurrence of disconnected states of consciousness and episodes of musculoskeletal contortions, leading him to classify “demonomania” as a form of hysteria [28]. Both Charcot and Janet considered the hysterical phenomena in their patients to largely represent neurodegenerative conditions and they sought to separate these conditions from their historical enmeshment in occult and superstitious beliefs [29,30,31].

Charcot was maligned for his flamboyant and theatrical public demonstrations of hysterical phenomena and his miraculous “cures” of hysterical displays, which allegedly contributed to epidemic manifestations of such phenomena [10,13,18]. He was further criticized for failing to consider potential contributions of malingering and suggestion in the production of hysterical phenomena, especially when hypnosis was used, and for promoting social contagion by housing hysterics and epileptics together [7,13,19]. He was also criticized for various other methodological shortcomings [7,32].

Despite being discredited by his peers, Charcot successfully paved the way for the influential work of Austrian neurologist Sigmund Freud. Freud spent a few months early in his career studying hysterical phenomena, especially hysterical seizures, under Charcot [31], using hypnosis with these patients. The classic case of Anna O., who was treated for a hysterical condition by Freud’s Austrian colleague Josef Breuer [33], exhibited dual personalities and episodes of amnesia, paralysis, aphonia, deafness, diplopia, visual hallucinations of snakes, memory disturbances, and loss of ability to speak her native language.

Freud is credited with having first introduced the concept of hysterical conversion, and he originally coined this term [29,34]. Freud emphasized psychological origins to hysterical conversion phenomena, in which ideas or memories too unpleasant for conscious awareness are repressed into the unconscious and “converted” into physical symptoms to solve unbearable psychological conflicts [20,26,30,33,34,35,36]. Freud’s work with hysteria formed the theoretical basis for the development of the field of psychoanalysis and the techniques he used to treat hysteria [27]. As Freud’s repression-based theory of hysteria gained popular support, Janet’s dissociation-based theory faded from prominence, falling into relative disuse for most of the next century [11].

In summary, this history reflects centuries of commingling of spirit possession, dissociation, hysteria, and conversion. Mainstream interest in spirit possession eventually faded, but major conversion and dissociative syndromes with dual multiple personalities have persisted to the present, with subsequent periodic spurts in reporting of these phenomena. While Charcot’s student Janet focused on dissociative aspects of hysteria (disturbances of conscious awareness involving amnesia and identity confusion), his other student Freud was most concerned with the conversion aspects of hysteria involving neurological complaints without medical basis in sensory and motor pathology [22]. Despite the apparent differences in these approaches, the separation between dissociative and conversion phenomena was uncertain even at the time, as evidenced by Janet’s belief that Freud had plagiarized his ideas [31]. Chodoff [29] pointed out that Freud’s hysteria was “symptomatically no different from hysteria described by Charcot and Janet” [29] (p. 1073). Breuer [37] concluded that the dissociative concept of “double conscience...is present to a rudimentary degree in every hysteria” [37] (p. 63), a phenomenon he termed “hypnoid.” At this point in history, dissociation and conversion were clearly still embedded within a unified concept of hysteria.

## 3. Dissociation, Conversion, and Hysteria in the American Diagnostic System for Mental Disorders

In 1909, the English physician Savill resurrected Sydenham’s description of hysteria from two centuries earlier. Savill described the syndrome as “manifested by an immense variety of nervous, neuromuscular, neuro-vascular, sensory, and other symptoms which may be referable to almost any organ or part of the body” and which are “unaccompanied, as a rule, by any obvious physical signs [or]…any gross or microscopic anatomical changes” [38] (p. 5). Later in that century, Savill’s characterization of hysteria was further affirmed in America by the Washington University psychiatry group in St. Louis, who established a set of criteria for hysteria known as the Perley-Guze checklist [39] based on seminal work by Briquet [23], Savill [38], and Purtell, Robins, and Cohen [40]. Their criteria were eventually published as part of the historical “Feighner criteria” [41] that strongly influenced the American diagnostic system. The Washington University group adopted the name “Briquet’s syndrome” to replace the older term “hysteria,” which had long since become heavily laden with pejorative connotations [40,42,43,44].

Briquet’s syndrome is a chronic, typically lifelong disorder that typically begins in the decade following puberty and occurs almost exclusively in women [45,46]. Its prevalence is approximately 1%–2% among general population women [45]. Patients with this disorder visit many physicians with a plethora of complaints lacking medical explanation or physiologic basis. Their medical histories are often dramatic and complicated. These patients tend to undergo extensive surgical procedures and invasive tests, often with complicated courses and poor outcomes. Briquet’s syndrome may seriously impair social and vocational functioning, and some patients are completely incapacitated by it [46]. There is no cure for this disorder and no specific psychotropic medication indicated for it, but with effective management, many of these patients can be stabilized. Treatment is ideally orchestrated by one physician who forms a supportive relationship with the patient to help redirect her from her many symptoms to address the many psychosocial issues typically accompanying this disorder. The ultimate goal of treatment is to prevent iatrogenic morbidity through protecting the patient from excessive medications, diagnostic procedures, and surgeries [45,46].

The criteria for Briquet’s syndrome defined the disorder as requiring a lifetime history of at least 25 clinically significant and medically unexplained symptoms from a list of 59 symptoms, represented in at least 9 of 10 organ systems, beginning by age 30. This characteristic presentation of recurrent symptoms in many different organ systems [47] has been described as a “polysymptomatic, polysyndromic” pattern [14,48]. Conversion symptoms are embedded in the criterion symptoms of Briquet’s syndrome and are commonly observed in these patients. Symptom presentations that are confined to medically-unexplained neurological symptoms are, however, considered to represent a conceptually related, but separate disorder classified as conversion disorder. Evidence to support the separation of these two syndromes is based on the different distinctive clinical presentations and very different longitudinal courses of these two syndromes [47].

The above history documents that in American psychiatry by the middle of the twentieth century, the syndrome of hysteria was firmly established and was defined as multiple recurrent unexplained physical symptoms presenting in many different organ systems [40]. The St. Louis criteria for the disorder and the name Briquet’s syndrome were routinely used in clinical practice and research for the next few decades at Washington University, a convention that subsequently spread to several affiliated sectors [49].

At the time of these historic developments in St. Louis, the American system of diagnostic criteria for mental disorders was evolving. The first edition of the *Diagnostic and Statistical Manual of Mental Disorders* (*DSM*) of the American Psychiatric Association [50] listed “dissociative reaction” together with “conversion reaction” in a section for “psychoneurotic disorders” that also included anxiety (e.g., “anxiety hysteria”) and depressive “reactions.” The text noted that dissociative reaction was formerly classified as a type of conversion hysteria. The second edition of the manual (*DSM-II*) [51] listed dissociation and conversion as two different types of “hysterical neurosis” within a section entitled “Neuroses” that also included a separate diagnosis of “depersonalization neurosis (depersonalization syndrome)” as well as a diagnosis of “hypochondriacal neurosis” [51] (p. 41). The manual endorsed a position that the “distinction between conversion and dissociative reactions should be preserved” [51] (p. 39).

After *DSM-II*, dissociation was largely absent from discussions of hysterical syndromes. The term “somatoform” was originally established to refer to physical symptom complaints without a medical basis, reminiscent of those represented in the older concepts of hysteria and Briquet’s syndrome. The first article listed in *PubMed* that includes the term “somatoform” was published in 1978, defining somatoform symptoms as “recurrent and multiple somatic complaints for which medical attention is sought but that apparently are not due to any physical disorder” [52] (p. 1501). *Somatic* symptoms, which refer to any physical symptom without regard to medical basis, are differentiated from *somatoform* symptoms, which are physical symptoms that are medically unexplained [53].

Historically, psychodynamic theory with its long-held assumptions of etiology of dissociation, conversion, and somatization provided the main process for distinguishing these syndromes and categorizing them separately from one another [54]. However, the basis of psychodynamic theory in the formulation of criteria for psychiatric disorders was formally abandoned 35 years ago beginning with the third edition of the *Diagnostic and Statistical Manual of Mental Disorders* (*DSM-III*) [55]. Thereafter, an atheoretical and agnostic approach to psychiatric diagnosis based on measurable and reliable features of disorders including characteristic symptoms, longitudinal course, and familiality came to dominate the American system of diagnostic criteria [56]. The phenomenological and atheoretical St. Louis criteria for Briquet’s syndrome were introduced into the American diagnostic criteria in *DSM-III* in a streamlined format. The diagnosis of somatization disorder as defined in *DSM-III* required a lifetime history of 14 of 37 possible symptoms, reduced from the requirement of 25 of 59 possible symptoms for the diagnosis of Briquet’s syndrome. The somatization disorder criteria did not specifically require distribution of symptoms throughout multiple organ systems as described in Briquet’s syndrome, even though the criterion symptoms of somatization disorder were listed in seven categories of symptom types. The name “somatization disorder” (not “Briquet’s syndrome”) was adopted in *DSM-III*, and this disorder anchored its somatoform disorders category. The *DSM-III* somatoform disorders category also included conversion disorder “(or hysterical neurosis, conversion type)” [55] (p. 244), psychogenic pain disorder, hypochondriasis “(or hypochondriacal neurosis)” [55] (p. 249), and atypical somatoform disorder [55].

North, Ryall, Ricci, and Wetzel [14] and Martin [57] have pointed out that the *DSM-III* criteria for somatization disorder included only the somatic symptoms and not the psychological symptoms of the Perley-Guze criteria for Briquet’s syndrome. These authors coined the term “psychoform” [14] (pp. 25–26) to characterize the psychological symptoms of Briquet’s syndrome that were not included in the *DSM-III* criteria for somatization disorder. In parallel with the term “somatoform” referring to symptoms that suggest (“-form”), but are not part of, an established *somatic* disorder, North and her colleagues introduced the term “psychoform” to describe symptoms that suggest but are not part of an established *psychiatric* disorder. Psychoform symptoms from the Perley-Guze criteria for Briquet’s syndrome that were not included in *DSM-III* criteria for somatization disorder included anxiety (nervousness, fears), depressive (low mood, crying, hopelessness, suicidal ideation), and psychotic (hallucinations) symptoms [14]. The term “psychoform” as defined by North *et al.* was subsequently applied by Wetzel, Guze, Cloninger, Martin, and Clayton [58] (p. 565) and has also appeared in several other academic publications [16,17,59,60].

*DSM-III* separated dissociative from somatoform disorders and grouped conversion with the somatoform disorders. The dissociative disorders section was placed immediately after the somatoform disorders section to reflect the recognition of a conceptual proximity of these two groups of syndromes. In the agnostic and atheoretical approach to American psychiatric diagnosis established with *DSM-III*, the grouping of conversion with somatoform phenomena emphasized the phenomenological association of physical symptom complaints. This convention displaced the causal assumptions of psychoanalytic theory that had previously driven the categorization of these syndromes [11]. The opening statement of the *DSM-III* dissociative disorders section stated that the essential feature of dissociative disorders involved “alteration in the normally integrative functions of consciousness, identity, or motor behavior” [55] (p. 253). The *DSM-III* dissociative disorders included psychogenic amnesia, psychogenic fugue, multiple personality, depersonalization disorder, and atypical dissociative disorder. The text explained that placement of depersonalization disorder in this section was controversial because depersonalization disorder, unlike all the other disorders in this section, did not involve memory disturbance.

The revised third edition of the *Diagnostic and Statistical Manual of Mental Disorders* (*DSM-III-R*) [61] made relatively minor changes to somatoform and dissociative disorders. Psychogenic pain disorder was renamed “somatoform pain disorder.” A new diagnosis of body dysmorphic disorder was added to the somatoform disorders. *DSM-III-R* described the essential feature of dissociative disorders as involving “alteration in the normally integrative functions of identity, memory, or consciousness,” [61] (p. 269) replacing motor behavior (a peripheral nervous system manifestation) with memory (a central nervous system manifestation) in this list, in an apparent efforts to further differentiate dissociative phenomena from somatoform and conversion disorders.

In the fourth edition of the *Diagnostic and Statistical Manual of Mental Disorders* (*DSM-IV*) [62] and its subsequent text revision (*DSM-IV-TR*) [63], the criteria for somatization disorder were further simplified to require only eight symptoms (from a list of 32 symptoms listed as examples) distributed among four symptom groups. Included among the examples of “pseudoneurological” symptoms from this list was amnesia, described as a dissociative symptom, in contrast to impaired coordination or balance, paralysis or localized weakness, difficulty swallowing or lump in throat, aphonia, urinary retention, hallucinations, loss of touch or pain sensation, double vision, blindness, deafness, and seizures, a collection of symptoms also referred to as conversion symptoms. Somatoform pain disorder was renamed “pain disorder.” At the same time, the diagnosis of psychogenic amnesia in the dissociative disorders section was renamed “dissociative amnesia” to be more consistent with the international criteria for psychiatric disorders. In *DSM-IV*, perception was added to the previous list of disrupted functions of consciousness, memory, identity disrupted as the essential feature of dissociative disorders. Psychogenic fugue was renamed “dissociative fugue.” Multiple personality disorder was renamed “dissociative identity disorder.” The section on factitious disorders was inserted between somatoform and dissociative disorders.

In the fifth edition of the *Diagnostic and Statistical Manual of Mental Disorders* (*DSM-5*) [64], somatoform disorders established in previous editions of the criteria were extensively reconceptualized into a new section of “somatic symptom and related disorders.” Somatization disorder was removed entirely. The major diagnosis in this section is now somatic symptom disorder, which requires one or more physical symptoms that cause distress or significant disruption of daily life. The prior somatoform disorders requirement that the symptoms be medically unexplained was not included in somatic symptom disorder criteria. Diagnoses of hypochondriasis, pain disorder, and undifferentiated somatoform disorder were dropped.

Conversion disorder was retained in *DSM-5* and it received a new subtitle: “functional neurological symptom disorder.” Diagnostic criteria for conversion disorder retained the medically unexplained requirement for symptoms to qualify for the diagnosis, requiring objective clinical evidence of internal inconsistency on neurological examination or incongruity with known neurological presentation of illness. The previous requirement that the symptom(s) must be associated with psychological conflicts or stressors, which invoked a theoretical etiology, was removed. The old name “conversion disorder, however, was preserved, even though the wisdom of keeping this name has been debated because of the theoretical and etiological vestiges of psychoanalytic theory it represents, which were ostensibly removed from the American diagnostic criteria in 1980. The prior requirement that the symptom or deficit not be intentionally produced or feigned has been removed from *DSM-5* criteria for conversion disorder, allegedly because this distinction is difficult or impossible to make; the text, however, states that definite evidence of intentionality or feigning would alternatively suggest diagnoses of factitious disorder or malingering. Additionally, *DSM-5* eliminated the factitious disorders section and moved its content into the section for somatic symptom and related disorders.

Although functional impairments considered central to the psychopathology of dissociative disorders have varied across editions of the American diagnostic manual, *DSM-5* describes dissociative disorders as broadly involving impairments in the integration of all of the following: consciousness, memory, identity, emotion, perception, body representation, motor control, and behavior, “potentially disrupting every area of psychological functioning” [64] (p. 291). (Motor functions were re-inserted, and emotion and body representation were added.) In *DSM-5*, the diagnosis of depersonalization disorder was changed to “depersonalization/derealization disorder” and dissociative fugue was added as a specifier for the diagnosis of dissociative amnesia.

The *DSM* classification system and its directional changes have had far-reaching ramifications for diagnostic practice. As noted by Paris [12], once the dissociative disorders had finally obtained a separate diagnostic category dedicated to them in the American diagnostic criteria in 1980, a following of believers emerged who fervently maintained that dissociative disorders are common, under-recognized, and very important. Around that time, a sudden upsurge in reported cases followed publication of the best-selling book *Sybil* [65] describing a dramatic case of multiple personality disorder with 16 alternate personalities. The popularity of this book and a subsequent Hollywood movie based on the book heralded a veritable epidemic of this disorder in the decades to follow [12,66]. The number of reported cases of multiple personality disorder in the world’s literature increased dramatically in the 1980s [14,67], with more cases discovered just in the first half of that decade than in the preceding two centuries [67,68]. These modern cases were concentrated in the United States and were contributed by a limited number of authors who extensively cross-referenced one another [14]. Not only did the numbers of reported cases expand rapidly, but the numbers of separate personalities reported in these cases also markedly increased [14].

More recently, Pope and colleagues [69] tracked the number of scientific articles on dissociative identity disorder and repressed memory, finding the emergence of a dramatic increase in the 1980s that spiked in the late 1990s and then plummeted to about one-fourth of the peak by the early 2000s. Over this same period, academic publication rates for 25 other psychiatric disorders were either constant or sustained gradual increases. It was concluded from these trajectories in academic publications that the dissociative disorders have not consistently garnered professional interest and acceptance over the long term, rather reflecting transitory fashions in the popularity of psychiatric diagnosis—described by Paris as a “psychiatric fad” [12] (p. 1078).

## 4. Continuing Controversies over Classification of Dissociative, Conversion, and Somatoform Disorders

The classification of dissociative disorders in the American diagnostic system for mental disorders fell out of line with the international diagnostic criteria in the late twentieth century. The ninth edition of the *International Criteria for Disease* (*ICD-9*) in 1978 [70] included dissociative (including “hysterical” amnesia and fugue and “dissociative” identity disorder), conversion (including “hysterical” blindness, deafness, paralysis, astasia-abasia, and “conversion hysteria or reaction”), and factitious disorders together in one single category. Separate categories were provided for somatoform disorders (the main diagnosis being somatization disorder with a subtitle of Briquet’s disorder), depersonalization disorder (including derealization disorder), and hypochondriasis. Thus, in *ICD-9*, conversion was included with dissociation and separated from somatization. The tenth edition of the international criteria (*ICD-10*) [71] also provided a category for both dissociative (including amnesia, fugue, stupor, and identity disturbance) and conversion (including “dissociative” motor disorder, aphonia, “dissociative” seizure/convulsion, “dissociative” sensory loss/deafness, and trance/possession) disorders together. As with *ICD-*9, a separate category was devoted to somatoform disorders (with somatization/Briquet’s disorder, hypochondriacal disorder, body dysmorphic disorder, and pain disorder). Thus, *ICD-10* retained the previous edition’s classification of conversion and dissociation together, separate from somatization.

Reviewing the very long world history of somatization, conversion, and dissociation, Bowman [11] pointed out that these disorders were closely intertwined under the common label of hysteria for nearly four millennia—until the late twentieth century. Bowman noted that the dissociative processes of Janet’s patients had included not only somatic disturbances of vision, hearing, speech, movement, and sensation but also psychological alterations of consciousness, memory, and identity. Bowman characterized the break of *DSM-III* from previous tradition with its separation of conversion from dissociation and grouping of conversion with somatoform disorders as “a scientific embarrassment for American psychiatry” [11] (p. 186). The move to fold conversion into somatization in the American diagnostic system, according to its detractors, fell out of step with the international criteria and detracted from the understanding of these disorders [11,20,72]. Spiegel [73] concluded that “dissociative disorders have never been comfortably integrated into psychiatric nosology” [73] (p. 261).

Several authors [11,66,72,74,75,76,77,78] have called for the reclassification of conversion as part of dissociative disorders in the American diagnostic system. Brown and colleagues [72] have further suggested the possibility of moving somatization along with conversion into the dissociative disorders category.

Along similar lines, Nijenhuis [79] recommended division of dissociation into somatoform and psychological components to reflect an appreciation that “The major symptoms of hysteria…involve *both mind and body*” [79] (p. 8); italics added). In this conceptualization, “somatoform dissociation” refers to symptoms phenomenologically involving physical functions of the body, and “psychological dissociation” refers to symptoms phenomenologically involving mental functions of memory, identity, and consciousness [65]. The inclusion of “dissociation” in both terms was chosen to clarify that both phenomena theoretically involve mental processes with disrupted integration of experiences, reactions, and functions, in both somatic and psychological domains [79,80]. In the Nijenhuis *et al.* conceptualization, symptoms of somatoform dissociation would include classic conversion symptoms such as anesthesia, analgesia, pain, blindness, deafness, and loss of motor control [66,79]. Symptoms of psychological dissociation would include dissociative amnesia, depersonalization, derealization, identity confusion, identity fragmentation, out-of-body experiences, altered time perception, loss of control, and mental absorption [81,82]. Nijenhuis later renamed psychological dissociation as “psychoform dissociation” [83] (p. 678); [84] (p. 982); [85], (p. 263). Nijenhuis [85] conceptualized “psychoform” and “somatoform” dissociative phenomena as “overlapping, but not identical manifestations of a common process” [85] (p. 271).

Kihlstrom [66] recommended subdivision of dissociative disorders into three categories. The first dissociative category in his conceptualization would involve memory abnormalities, representing the disorders currently classified in the American criteria as dissociative. The second dissociative category would involve sensory abnormalities, including some symptoms currently classified as conversion, such as psychogenic blindness, deafness, and anesthesia. The third dissociative category he proposed would involve motor abnormalities, including some symptoms currently classified as conversion, such as psychogenic paralysis and aphonia.

## 5. Extensive Phenomenological Overlap of Dissociative, Conversion, and Somatoform Disorders and Related Psychopathology

A practical reason for grouping conversion and somatoform disorders together in the American diagnostic system has been to emphasize the importance of determining that the symptoms of both types of disorders do not simply represent the pathology of an established medical or neurological condition [72]. Classifying somatization and conversion syndromes together within a single somatoform disorders category provides additional utility by prompting clinicians to also consider other medically-unexplained somatic complaints in patients presenting with conversion symptoms, many of whom will be found to meet full criteria for somatization disorder [86].

Recent recommendations to reclassify conversion within dissociative phenomena stem from theoretical assumptions that the etiology of conversion, like dissociation, involves abnormalities of integration of consciousness [11]. This reasoning may potentially explain why the American diagnostic system, with its atheoretical and agnostic approach to diagnosis, resisted this move. Further evidence presented in support of arguments for joining conversion and dissociation into a single category is the extensive comorbidity of these phenomena within the same individuals and the similarity of other features of patients with these disorders [11]. The American diagnostic manual, however, while retaining separation of these disorders, also acknowledges this extensive comorbidity and clinical overlap between them [64].

The degree of overlap among dissociative, somatoform, and conversion phenomena within patients has complicated efforts throughout history to categorize these disorders. Additional psychiatric comorbidities demonstrated in association with these disorders, especially borderline personality disorder, have further confused efforts to categorize these disorders.

A review by Brown and colleagues [72] of studies of dissociative disorder found substantial somatoform and conversion symptom comorbidity. Dissociative identity disorder is especially associated with numerous psychiatric comorbidities. An extensive review of multiple personality disorder by North *et al.* [14] demonstrated abundant evidence of comorbidity with somatization disorder (33%–100%), conversion disorder (generally > 50%), and borderline personality disorder (about 50%–70%), with three or four comorbid disorders on average. Patients in this review were found to present so many symptoms of psychotic, depressive, manic, anxious, phobic, eating, substance use, antisocial, and hyperactivity disorders that the authors stated that multiple personality disorder “may be best conceptualized as a polysymptomatic, polysyndromic disorder” [14] (p. 48) similar to established conceptualizations of somatization disorder. Kluft [87] noted that dissociative identity disorder is rarely seen in the absence of other psychiatric disorders. Putnam and colleagues [88] observed that multiple personality disorder mimics not only the range of psychiatric syndromes but also many physical illnesses.

Pseudoneurological or conversion symptoms have long been included as part of diagnostic criteria for hysteria and somatization disorder, demonstrating a clear recognition of the frequent co-occurrence of these symptoms in patients with these disorders. In the criteria for Briquet’s syndrome, one of the ten symptom groups included 11 medically unexplained neurological symptoms (blindness, paralysis, anesthesia, aphonia, fits or convulsions, unconsciousness, amnesia, deafness, hallucinations, urinary retention, and trouble walking) as well as “other unexplained ‘neurological symptoms’” [41] (p. 60). *DSM-IV-TR* criteria for somatization disorder required at least one pseudoneurological symptom from a list of 13 symptoms (impaired coordination or balance, paralysis or localized weakness, difficulty swallowing or lump in throat, aphonia, urinary retention, hallucinations, loss of touch or pain sensation, double vision, blindness, deafness, seizures, amnesia, and loss of consciousness other than fainting) [63]. The classic study of 50 patients with somatization disorder by Purtell, Robins, and Cohen [40] found a high prevalence of conversion symptoms, including blindness in 20%, paralysis in 33%, lump in throat in 74%, loss of voice in 45%, paralysis in 33%, and paresthesias in 80%. Of note, trance states (considered by Guze and Perley [47] to represent conversion symptoms yet are currently classified as dissociative phenomena in *DSM-5*) were also reported in 42% of their patients, but no further information on dissociative phenomena was provided.

A review by Brown and colleagues [72] of studies of patients with conversion disorder found that one-third to one-half of the patients were identified to have a comorbid dissociative disorder. Other studies have found psychiatric comorbidities in 90% or more of patients with conversion disorder, including nearly one-half with dissociative disorder [89,90]. Patients with both conversion and dissociative disorders were especially likely to also have borderline personality disorder [89]. One study of patients with pseudoseizures found a lifetime history of comorbidity with somatoform disorder in 98%, dissociative disorders in 93%, and borderline personality disorder in 62% [91].

Somatization disorder, defined as a disorder of many symptoms of many different types, both physical and mental [17], provides fertile soil for patients to be diagnosed with additional medical and psychiatric disorders. In the Epidemiologic Catchment Area (ECA) household study of the prevalence of psychiatric disorders in America [92], 100% of individuals diagnosed with somatization disorder also met criteria for another psychiatric disorder. Schizophrenia and anxiety and depressive disorders were significantly associated with the diagnosis of somatization disorder. The ECA study did not assess conversion disorder, dissociative disorders, or borderline personality disorder. A study of patients with somatization disorder by Liskow and colleagues [93] found that 99% of these patients met diagnostic criteria for other psychiatric disorders, and the mean number of comorbid disorders in these patients was 4.3. The high rates of diagnostic comorbidity with schizophrenia found in patients with somatization disorder [93,94] coupled with a noteworthy absence of schizophrenia among their relatives [93] led Martin [57] to question the interpretation of psychotic symptoms in the context of somatization disorder.

The inclusion of hallucinations, depressive symptoms, and anxiety as defining symptoms of Briquet’s syndrome (consistent with the term “psychoform” first described by North *et al.* in 1993) likely reflected a formal recognition of the importance of these symptoms along with somatoform symptoms in defining the disorder. Martin [57] remarked on the high prevalence of psychiatric symptoms in patients with somatization disorder, pointing out that anxiety and depressive symptoms have been traditionally embedded in Briquet’s syndrome criteria and prominently featured in classic presentations of somatization disorder. Schwartz *et al.* [94] found that many patients with somatization disorder described depressive symptoms (86%), psychotic symptoms (68%), and nervousness (75%). The frequently observed comorbidities of other psychiatric symptoms and disorders with somatization disorder defined without psychoform symptoms in the American criteria suggest a real association rather than an artifact created by psychiatric symptoms embedded in the diagnostic criteria.

The frequency of presentation of other psychiatric symptoms in patients with somatization disorder has prompted further investigation of this association. DeSouza and colleagues [95] reported that patients with somatization disorder had significantly greater numbers of depressive symptoms even than patients diagnosed with major depressive disorder. Consistent with this observation, North and her research team [59] found that patients with somatization disorder acknowledged significantly as many or more current and lifetime symptoms of other psychiatric disorders compared to patients with other psychiatric disorders but without somatization disorder, although almost none of the somatization disorder patients met diagnostic criteria for these other diagnoses. Patients in this study with cluster B personality disorders (predominantly borderline personality disorder) without somatization disorder also endorsed as many psychiatric symptoms of these other disorders as patients with somatization disorder, significantly more than patients with other Axis I psychiatric disorders.

Comorbidity of somatization disorder and conversion disorder with borderline personality disorder is well documented [14]. Borderline personality disorder is characterized in current diagnostic criteria as a pattern of instability in interpersonal relationships, self-image, and emotional regulation, with marked impulsivity [64]. Studies of patients with borderline personality disorder [96,97,98] have found that 96%–100% of these patients have psychiatric comorbidities, with a mean of 3.4–5.1 comorbid disorders [96,98]. In one of these studies [96], nearly two-thirds (62%) of the patients also met diagnostic criteria for Briquet’s syndrome; additionally, the comorbidity with somatization disorder was higher using *DSM-IV* criteria than with *DSM-III* criteria.

Hudziak’s group [96] was impressed by the similarities in the criteria for borderline personality disorder and descriptions of the characteristics of patients with Briquet’s syndrome, leading them to consider the possibility that Briquet’s syndrome might actually constitute a subset of borderline personality disorder. They further reasoned that the apparent comorbidity of borderline personality disorder and Briquet’s syndrome may represent not true comorbidity of two distinct disorders, but rather a reflection of the shared conceptual criteria for these diagnoses.

Hudziak and colleagues [96] puzzled over a finding in their study that they did not expect. Previous research had demonstrated that a higher proportion patients with somatization disorder met criteria for somatization disorder criteria than the proportion of patients with Briquet’s syndrome found to meet somatization criteria [99], suggesting that the criteria for Briquet’s syndrome are more restrictive than the criteria for somatization disorder in clinical use. Hudziak’s group [96], however, found Briquet’s syndrome to be more prevalent than somatization disorder in their patients with borderline personality disorder. This seemingly inconsistent finding likely reflects the inclusion of psychoform symptoms in the definition of Briquet’s syndrome but not in the definition of somatization disorder and is resonant with the characteristic comorbidity of symptoms of many psychiatric disorders found among patients with borderline personality disorder [59]. Hudziak and colleagues [96] commented on the amount of shared clinical characteristics between Briquet’s syndrome and borderline personality disorder.

Remarkable overlaps of clinical features among patients with dissociative, somatoform, conversion, and borderline personality syndromes have been described elsewhere [11,17,100]. Patients with these disorders have been variously described as having in common a female preponderance; a multiplicity of symptom complaints; chronic course of illness; vague, circumstantial, imprecise, and exaggerated descriptions of their symptoms; dramatic style of presentation; suggestibility and hypnotizability; voluminous of symptoms of many types; extensive comorbidities; psychotic-like symptom presentations; emotional instability and difficulties with affect regulation; impulsivity; suicidal ideation and attempts; marked and persistent identity disturbance; intense and volatile personal relationships; stormy marital histories; chaotic family backgrounds; and histories of childhood neglect and abuse [14,17,19,40,57,96,100,101,102].

Further evidence of similarities among patients with these disorders has been demonstrated through psychological testing. Nearly identical average profiles in patients with somatization disorder and dissociative identity disorder have been demonstrated using the original version of the Minnesota Multiphasic Personality Inventory (MMPI) [14]. Similarities in the profiles included: (1) an inverted V pattern on the L, F, and K scales and F scales above the usual validity cutoff, indicating a help-seeking, faking, or exaggeration style; (2) the “characterologic V” profile suggesting a personality disorder; (3) a floating profile with T-scores of 70 or more on most of the clinical scales, indicating complaints in almost every domain of psychological functioning; and (4) the “schizophrenia” scale was the consistently highest-scoring scale. Patients with borderline personality disorder demonstrate remarkably similar MMPI findings: when the average MMPI results of patients with somatization disorder, dissociative identity disorder, and borderline personality disorder are overlaid on a single graph, the profiles of the different disorders are nearly identical [14]. This MMPI evidence suggests that somatization disorder, dissociative identity disorder, and borderline personality disorder share much of their psychopathological material in common.

## 6. A New Conceptualization of Disorders Formerly Classified as Hysteria and Phenomenologically Related Disorders

Together, the extensive phenomenological overlap, similarities of presentation, and shared psychological material among somatoform, conversion, dissociative, and borderline personality disorders suggest that these disorders share a common axis of psychopathology and reveal fundamental problems with the current understanding of these disorders. The historical classification of these disorders based on theoretical assumptions of the psychopathology involved is inadequate to current need. A new conceptualization of these disorders is needed. A logical question is what determines how patients present along this axis with their specific psychopathology? Also, why do some patients present with an array of psychological symptoms but not physical symptoms, others present with single pseudoneurological symptoms, others present with the full array of physical and psychological symptoms, and yet others present with complex dissociative syndromes such as multiple personalities?

A common feature of all of these syndromes is that the presentation of symptoms does not follow established patterns of disease presentation or rules of anatomy and physiology. For example, areas of sensory anesthesia do not follow dermatomal distribution, motor paralysis does not follow the anatomy of motor nerve supply, non-epileptic seizures lack demonstrable brain waves of epilepsy, and psychiatric symptoms do not present with characteristic symptom clusters and patterns of known psychiatric disorders. Reynolds has explained that patients have their own ideas of how pathology works: they “have their own mental conceptions of right and left, of how motor or sensory function is distributed in a limb, of the function of muscle agonists and antagonists, and of the motor components of a seizure, which are quite different to those of a neurologist” [54] (p. 253).

**Figure 1:**
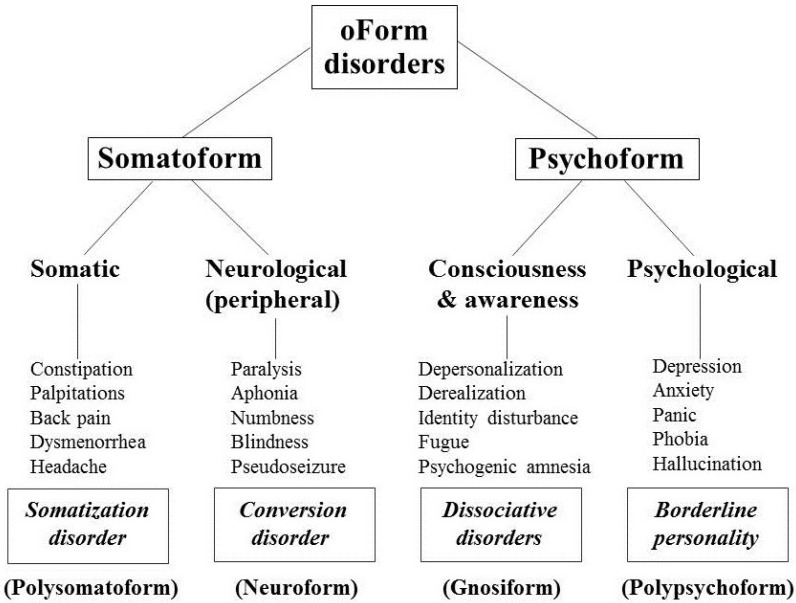
Schematic conceptualization of psychiatric disorders historically classified as hysteria and phenomenologically related psychopathology.

In the domains of shared psychopathology along the axis of somatization/conversion/ dissociation/borderline disorders, the psychopathology of some patients is largely confined to neurologically inconsistent motor or sensory symptoms, which are classified as conversion disorder. Other patients present symptoms throughout the body’s organ systems (often including conversion symptoms), placing the psychopathology within the category of somatization disorder; somatization disorder by definition subsumes the diagnosis of conversion disorder, which is not diagnosed if somatization disorder is present [63]). Patients presenting with syndromes involving multiple personalities, fugue states, and dissociative amnesia that involve higher-level brain integrative functions of identity, consciousness, and memory are considered to have dissociative disorders. Patients presenting with many psychiatric symptoms that suggest but are not part of several psychiatric disorders may well meet borderline personality disorder criteria. Figure 1 provides a schematic illustration of the relationships of these disorders based on the type of psychopathology exhibited. In this schematic diagram, “somatoform” syndromes include somatization and conversion disorders which present with physical symptoms of medical illnesses that the patient does not have: either throughout the body (somatization) or expressed in the sensory and motor peripheral nervous system (conversion). Dissociative and borderline personality disorders are classified as “psychoform” because they present with psychiatrically-based symptoms suggesting either central nervous system abnormalities of awareness and consciousness (dissociation) or symptoms of numerous psychiatric disorders for which the patient does not qualify (borderline personality).

This proposed categorical organization allows for some diagnostic overlap, based on the range of reported symptoms. Some patients with somatization or conversion disorders may also meet criteria for comorbid borderline personality or dissociative disorders. Some patients with borderline personality disorder may also meet criteria for somatization, conversion, or dissociative disorders. Patients with dissociative disorders might also meet criteria for any of the other disorders. Future use of this conceptual model may need to consider development of exclusion criteria to further differentiate its components.

Because the classification of disorders formerly conceptualized as hysterical involves two main categories of somatoform and psychoform symptoms, a new term, “oForm,” is suggested to refer to these categories of psychopathology. This term is descriptive and circumvents the longstanding pejorative term “hysteria” formerly previously used as the name for the psychopathology of these syndromes [39,41,42,43] and which has also been used to describe many different syndromes.

Other changes in the nomenclature for these types of psychopathology are warranted. A descriptive term for syndromes involving complaints of many physical symptoms that suggest but are not part of demonstrable medical disorders might be “polysomatoform,” reflecting the polysymptomatic, polysyndromic nature of this psychopathology. This term distinguishes this syndrome from the new DSM-5 entity of somatic symptom disorder that has replaced the former somatoform category and the diagnosis of somatization disorder which, unlike somatic symptom disorder, has been well validated by established methods of diagnostic validation [16] and thus merits retention in diagnostic systems of classification. A change in the name for conversion disorder is long overdue in the American system of psychiatric diagnosis that has distanced itself from theoretical definitions of these disorders since *DSM-III* in 1980, because the name “conversion” invokes a theoretical construct of psychic conflicts being converted into physical symptoms. A more descriptive name for symptoms and syndromes of this type might be “neuroform,” because these symptoms suggest neurological disorders that are not consistent with the patient’s symptoms.

Similarly, the traditional name “dissociation” refers to a theoretical concept of splitting of mental functions. A more descriptive name for psychiatric symptoms and syndromes involving functions of consciousness and awareness might be “gnosiform.” Finally, the name “borderline” historically evolved from the belief that the disorder represented a borderline syndrome of schizophrenia did not prove to be true, prompting the need for a more phenomenologically descriptive name for this type of disorder. A possible new name “polypsychoform” reflects these patients’ many psychiatric symptoms that suggest but are not part of established psychiatric disorders. These suggested new labels are included in the conceptual scheme in Figure 1.

## 7. Conclusions

The classification of disorders formerly known as hysteria and phenomenologically related disorders has long been contentious and unsettled. Examination of the long history of the conceptual difficulties, which remain inherent in existing classification schemes for these disorders, can help to address the continuing controversy. This article has reviewed the history of these disorders and their interrelationships with one another to clarify how the classification of these disorders over time has led to the current classification of these disorders. This review revealed that conceptual revision to the current classification of these disorders is needed. A new phenomenologically-based classification scheme for these disorders is proposed that is more compatible with the agnostic and atheoretical approach to diagnosis of mental disorders used by the current classification system.
